# Hyaluronan-Based Three-Dimensional Microenvironment Potently Induces Cardiovascular Progenitor Cell Populations

**DOI:** 10.1155/2013/752620

**Published:** 2013-09-03

**Authors:** Jessica M. Gluck, Jennifer Chyu, Connor Delman, Sepideh Heydarkhan-Hagvall, W. Robb MacLellan, Richard J. Shemin

**Affiliations:** 1 Cardiovascular Tissue Engineering Laboratory, Department of Surgery, David Geffen School of Medicine, University of California, Los Angeles, 10833 Le Conte Avenue, 62-151 CHS, Los Angeles, CA 90095-1741, USA; 2 Cardiovascular Research Laboratory, Departments of Medicine and Cardiology, David Geffen School of Medicine, University of California, Los Angeles, 675 Charles E. Young Drive South, MRL 3645, Los Angeles, CA 90095, USA; 3 Department of Medicine and Cardiology, University of Washington, 850 Republican Street, Brotman Room 432, Seattle, WA 98109, USA

## Abstract

The relationship between stem cell niches *in vivo* and their surrounding microenvironment is still relatively unknown. Recent advances have indicated that extrinsic factors within the cardiovascular progenitor cell niche influence maintenance of a multipotent state as well as drive cell-fate decisions. We have previously shown the direct effects of extracellular matrix (ECM) proteins and have now investigated the effects of dimension on the induction of a cardiovascular progenitor cell (CPC) population. We have shown here that the three-dimensionality of a hyaluronan-based hydrogel greatly induces a CPC population, as marked by Flk-1. We have compared the effects of a 3D microenvironment to those of conventional 2D cell culture practices and have found that the 3D microenvironment potently induces a progenitor cell state.

## 1. Introduction

Cardiovascular disease is one of the leading causes of morbidity and mortality in the United States. Conventional therapies include lifestyle changes, pharmacological and surgical intervention, and ultimately transplantation. Recent research has focused on recreating cardiovascular development *in vitro* to better understand heart tissue development and cardiovascular disease progression. By using tissue-engineering principles, we can build a better *in vitro* model.

The mammalian heart is believed to originate from a common progenitor cell. Cardiovascular progenitor cells (CPCs) further differentiate into three main cardiovascular lineages: vascular endothelial cells, vascular smooth muscle cells, and cardiomyocytes. Using our murine embryonic stem (mES) cell model, we have isolated CPCs using Flk-1 (also known as KDR), a cell surface marker for VEGF-R2. Endogenous CPCs are found in anatomical clusters in the developing right atria, ventricle, and outflow tract. These clusters are suggestive of a stem cell niche. Niches are three-dimensional (3D microenvironments that regulate self-renewal, proliferation, and differentiation through cell-cell and cell-matrix interactions [1, 2]. It is therefore very important to understand the role of the native extracellular matrix (ECM) that surrounds the CPC niche.

In the absence of signals from the surrounding ECM, cells can undergo apoptosis. Under cardiovascular disease, chronic volume overload leads to chamber remodeling. Various studies have shown the importance of ECM support necessary for angiogenesis and cardiovascular physiology. However, the exact nature of the ECM’s role during cardiac development remains unknown.

Hyaluronic acid (HA) is one of the major components found in ECMs throughout the body. It is a naturally derived, linear, and high molecular weight polymer with viscoelastic properties [3]. Additionally, HA has been shown to be involved in many biological processes, including wound repair, inflammation, and metastasis. The carboxyl group of HA is a major target site for further derivatization. HA-based hydrogels can be particularly useful to encapsulate various cell types to determine the effects of a three-dimensional microenvironment.

Previously, we have established that the ECM protein collagen IV induces a greater amount of CPCs than other proteins [2]. Additionally, incorporating a modified 2D substrate, a nanofibrous scaffold, into the culture system further induces a CPC population [4]. In order to further examine the full effects of a 3D microenvironment, we will develop a 3D *in vitro* culture system using a HA-based hydrogel.

## 2. Materials and Methods

### 2.1. Mouse ES Cell Culture.

Murine ES v6.5 cells were purchased from Open Biosystems and maintained in an undifferentiated, feeder-free state in leukemia inhibitory factor (LIF) supplemented medium (Knockout Dulbecco’s modified Eagle’s medium, Invitrogen, Carlsbad, CA, USA) with 15% ES-FBS (Invitrogen), 0.1 mM *β*-mercaptoethanol, 2 mM L-glutamine (Invitrogen), 0.1 mM nonessential amino acids (Invitrogen), 1000 U/mL recombinant LIF (Chemicon, Temecula, CA, USA), and 2 mM HEPES (Invitrogen). Cells were cultured on gelatin-coated (0.1% gelatin in PBS, coated for 2 h at 37C) T-75 f lasks at 37^∘^C, 5% CO_2_ in a humidified incubator. Cells were passaged every 2–3 days to maintain an undifferentiated state.

For initial differentiation assays, mES cells were introduced to collagen IV flasks (BD Biosciences, San Jose, CA USA) and maintained in α-minimum essential medium (α-MEM) (Invitrogen) supplemented with 10% ES-FBS (Invitrogen), 0.1 mM *β*-mercaptoethanol, 2 mM L-glutamine (Invitrogen), 0.1 mM nonessential amino acids (Invitrogen), and 2 mM HEPES (Invitrogen). After five days, cells were harvested for FACS analysis, fixed for immunostaining, or RNA was collected for PCR analysis.

### 2.2. Hydrogel Formation.

HA-based hydrogels were purchased from Glycosan Biosystems (subsidiary of BioTime, Alameda, CA, USA) and cross-linked with a PEGSSDA cross-linker. The hydrogel consists of a hyaluronan backbone and thiolated gelatin. The PEGSSDA, crosslinker allows for easy dissolution of the hydrogel at various time points. To encapsulate mES cells in the hydrogel microenvironment, the manufacturer’s protocol was followed. Briefly, the two components of the hydrogel backbone were dissolved in the provided diH_2_O and allowed to homogenize for 30 min on a rocker plate. mES cells were collected from the culture flask and centrifuged. The cell pellet was then resuspended in the hydrogel backbone components. The cross-linking agent, PEGSSDA is then introduced at a 4 : 1 ratio to the backbone components. Gelation occurs within 30 minutes from the cross-linker introduction. Equal volume of media (α-MEM) is added to the hydrogel after 2 h. In order to dissolve the hydrogel for further cellular analysis after 5 days, a reducing agent is added to the hydrogel. 40 mM L-glutathione solution is added directly to the hydrogel to break up the disulfide bonds formed from the cross-linking agent. After 1–2 hrs, the hydrogel-L-glutathione mixture is collected and centrifuged to obtain a cell pellet. The cells are then processed for FACS analysis, RNA collection, or cytospin imaging.

### 2.3. Live/Dead Cell Assay.

Live/Dead cytotoxicity assay was performed using the kit purchased from Invitrogen specific for mammalian cells. The manufacturer’s recommended protocol was followed. Briefly, a solution of sterile PBS, with 1 : 1000 calcein AM and 1 : 500 ethidium homodimer-1 was made. Cells were washed thoroughly with sterile PBS and the live/dead solution was added to the dish. Imaging immediately followed.

### 2.4. Fluorescence-Activated Cell Sorter Analysis.

Cells were harvested from both standard 2D and 3D (hydrogel) cultures, pelleted via centrifugation, washed in PBS supplemented with 1% ES-FBS and 2% BSA, and stained with purified rat anti-mouse Flk-1 antibody (BD Biosciences). The cells were gated by forward scatter (FSC) versus side scatter (SSC) to eliminate debris. A minimum of 10,000 events were counted for each analysis. All analyses were performed using a Becton Dickinson FACScan analytic flow cytometer (BD Biosciences) with FCS Express software (DeNovo Software, Thornhill, Ontario, Canada) at the UCLA Flow Cytometry Laboratory.

### 2.5. Quantitative Real-Time Polymerase Chain Reaction (PCR).

Total RNA was extracted from cells using TRI Reagent (Sigma-Aldrich) according to the manufacturer’s instructions. First-strand cDNA was generated from RNA by using the QuantiTect Reverse Transcription kit (Omniscript, Qiagen, Valencia, CA, USA). Real-time quantitative PCR was conducted using the ABI PRISM 7900 Sequence Detection System (TaqMan, Applied Biosystems, Carlsbad, CA, USA) with the UCLA GenoSeq Core. PCR amplicons were detected by fluorescent detection of SYBR green (QuantiTect SYBR green PCR kit, Qiagen). Data was analyzed using the ΔΔCt method, using ribosomal protein S26 as a stable reference gene for normalization control. Primers used were purchased from Qiagen (QuantiTect Primer Assay).

### 2.6. Immunofluorescent Staining.

mES cells at both undifferentiated and partially differentiated states were fixed using 4% paraformaldehyde in PBS for 20 min and rinsed with PBS. All samples were then washed with 0.05% Tween-20 in PBS three times, 5 min each, followed by a 15 min wash of 0.1% Triton-X in PBS. Three more washes of 0.05% Tween-20 in PBS for 5 min each followed. A blocking solution of 1% BSA and 2% goat serum in PBS was used on the cells for 30 min followed by incubation with the primary antibody overnight at 4^∘^C. The samples were then washed in PBS. A secondary antibody with Alexa Fluor 488 or 546 conjugation was then applied and incubated at room temperature for 30 min. After several washes of fresh PBS, cells were then counterstained with 4′−6-diamidino-2-phenylindole (DAPI) followed by anti-ProLong Gold antifade mounting medium (Molecular Probes, Carlsbad, CA USA). Digital images were acquired using a Leica DM IRB inverted microscope system equipped with 20x (0.40 numerical aperture (NA)) and 40x (0.75 NA) objective lenses (Leica Microsystems Inc., Bannockburn, IL, USA).

### 2.7. Statistical Analyses.

All results are presented as mean values ± standard error of mean (SEM). Statistical significance was assessed by *t*-test or ANOVA. *P* < 0.05was defined as statistically significant.

## 3. Results

### 3.1. Cells Maintained in HA-Hydrogel Survive.

**FIGURE 1:**
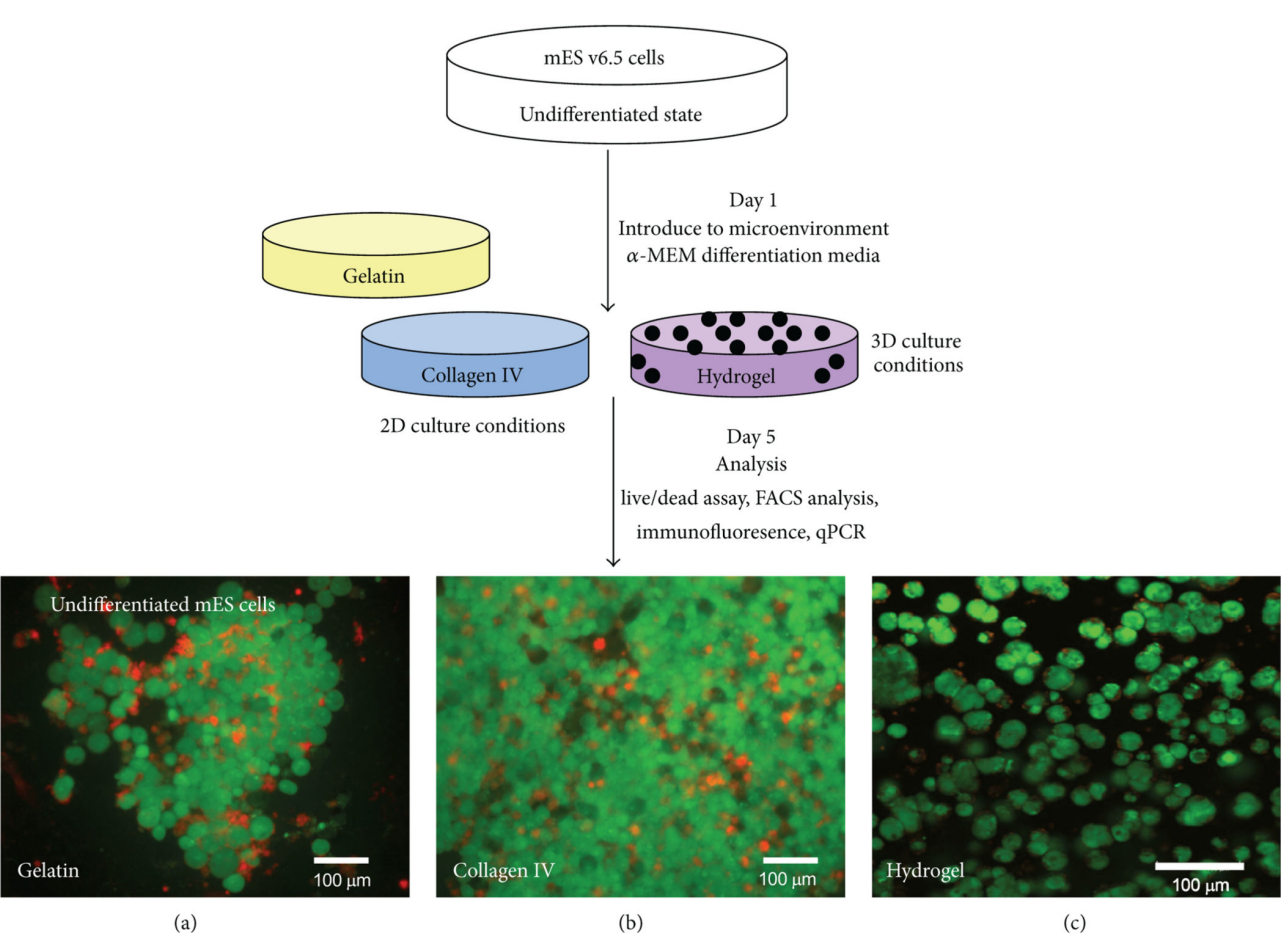
Schematic of experimental design. mES cells are cultured on either 2D or 3D conditions in differentiation media for five days. mES cells are then evaluated via a cytotoxicity assay, fixed for immunofluorescence, or harvested for FACS analysis and PCR. mES cells on 2D culture conditions with gelatin (undifferentiated) (a) or collagen IV (b) show a high amount of live (green) cells compared to dead cells (red) at 5 days. mES cells encapsulated in the Hystem-C hydrogel (c) also show a high number of live cells.

Hyaluronan hydrogels have been studied previously [5, 6], and in particular, Hystem-C hydrogels have been characterized previously [7, 8]. We have further investigated the biocompatibility of the encapsulated microenvironment of the hydrogel with mES cells. Using a live/dead cytotoxicity assay, we can see in Figure 1 that the environment of the hydrogel supports mES cell growth. We did observe a slower proliferation rate (data not shown) of cells encapsulated within the hydrogel as compared to cells maintained on gelatin- (for control) and collagen IV-coated dishes in 2D culture conditions, yet this was similar to the proliferation rates we observed previously when cells were cultured on nanofibrous scaffolds [2, 4]. Additionally, we observed different morphologies of cells in 2D culture versus 3D culture. Cells supported in 2D culture (Figure 1) exhibit more of a cluster or colony phenotype, characteristic of stem cell culture, while cells encapsulated in the 3D hydrogel (Figure 1(c)) are maintained in a more single-cell suspension-like phenotype. We also found a larger number of cells in the 2D culture dishes compared to the cells suspended in the hydrogel, which is evident from the images in Figure 1.

### 3.2. CPC Induction in Both 2D and 3D Microenvironments.

We have previously shown that collagen IV in 2D culture conditions induces a greater CPC population and that the addition of an electrospun scaffold further enhances induction of a Flk-1^+^ CPC population [2, 4]. While electrospun scaffolds can be classified as a 3D microenvironment, it can take mES cells up to seven days to migrate to the interior of the scaffold (data not shown). In order to investigate the true 3D effects, we encapsulated mES cells in the Hystem-C hydrogel system. After 5 days, we isolated cells for FACS analysis and checked for CPCs using the Flk-1 surface marker.

**FIGURE 2:**
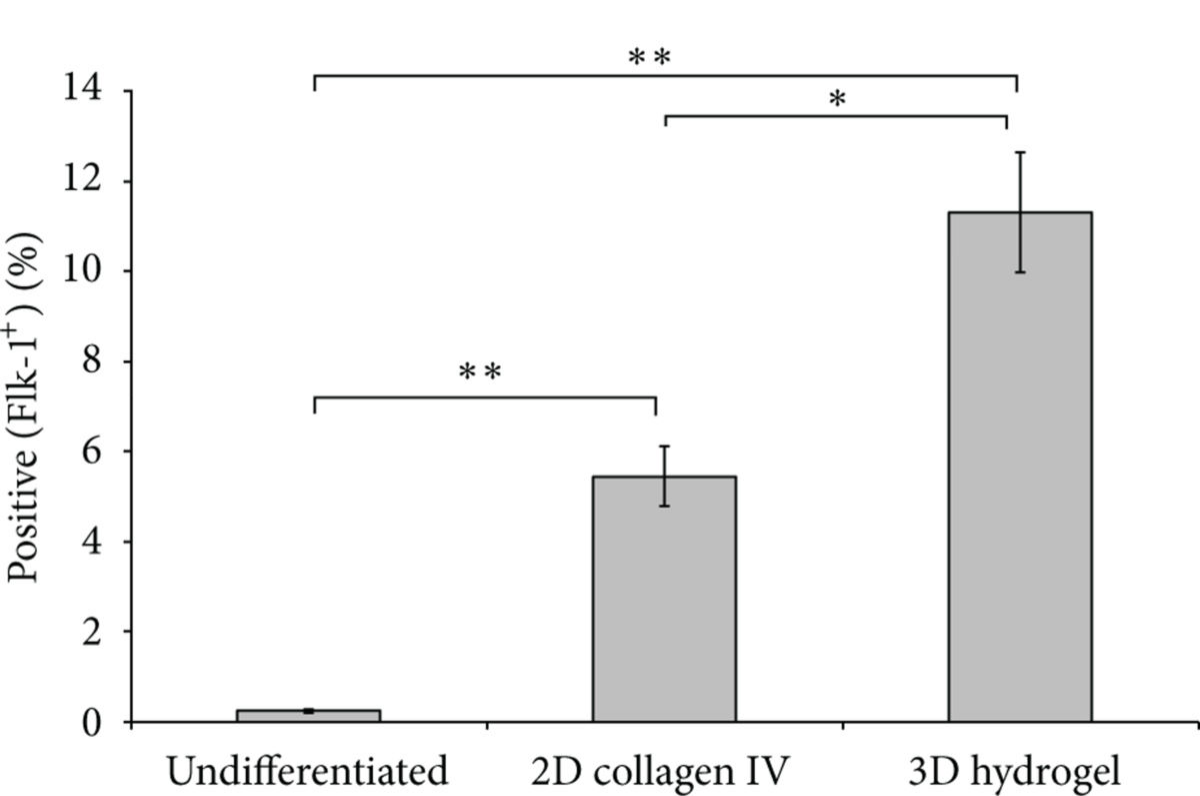
FACS analysis of Flk-1^+^ CPC population. FACS analysis of Flk-1^+^ cells from undifferentiated mES cells. mES cells cultured on collagen IV-coated 2D dishes and encapsulated in the 3D hydrogel were harvested for FACS analysis after five days in differentiation media. ^∗^*P* < 0.005, ^∗∗^*P* < 0.0001.

We found that the addition of the 3D microenvironment greatly enhances CPC induction (Figure 2). After 5 days in differentiation media, undifferentiated mES cells (0.24 ± 0.04% Flk-1^+^) expressed less than 1% Flk-1^+^ cells. Collagen IV, the protein previously shown to have the highest capacity to induce a Flk-1^+^ CPC population in 2D conditions [2], had 5.45 ± 0.66% Flk-1^+^ cells, while 11.32 ± 1.34% cells encapsulated in the Hystem-C hydrogel expressed the CPC marker. The hydrogel environment induced a significantly higher population of CPCs compared to both undifferentiated mES cells and those cultured on collagen IV-coated 2D dishes.

**FIGURE 3:**
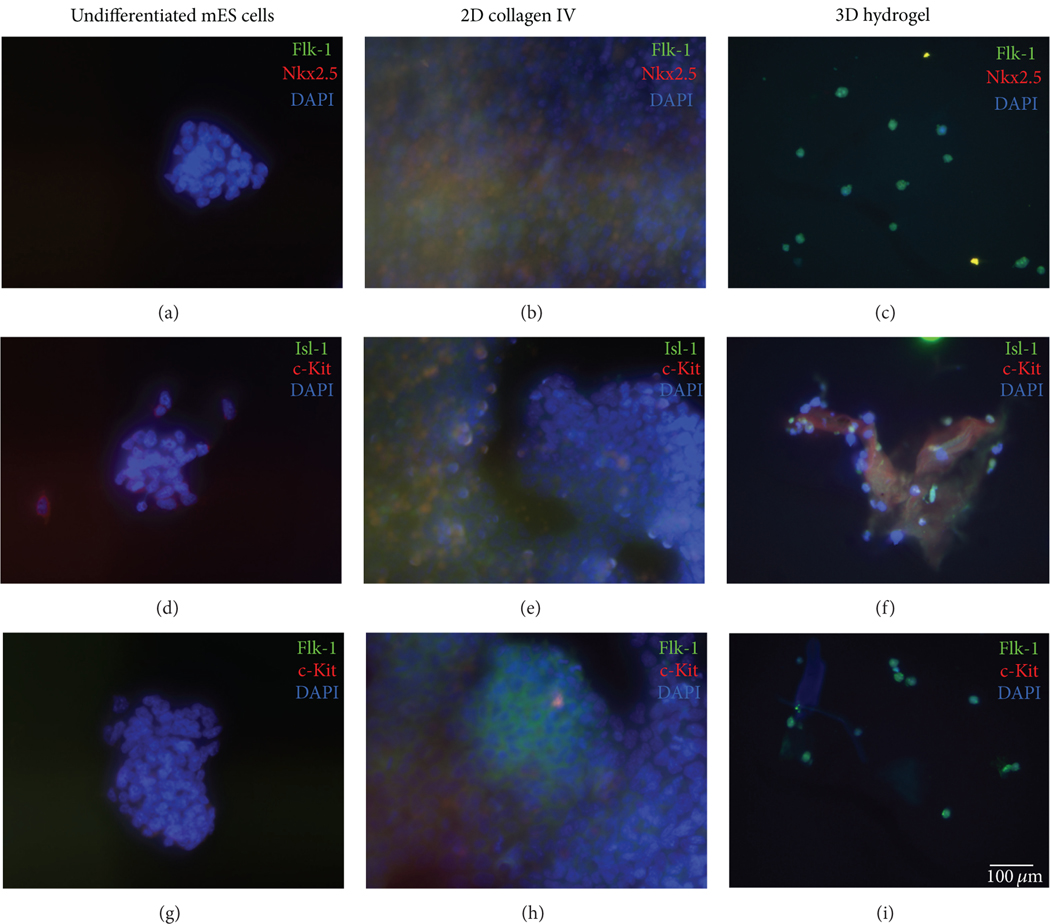
Immunofluorescence of CPCs. mES cells cultured in 2D culture conditions were immunostained for CPC markers after five days in differentiation media. Undifferentiated mES cells are shown in the first column of panels ((a), (d), and (g)). Cells cultured on 2D collagen IV-coated dishes are shown in the middle column of panels ((b), (e), and (h)). Cells encapsulated in the 3D hydrogel are shown in far right column of panels ((c), (f), and (i)). CPC markers Flk-1 (green) and Nkx 2.5 (red) are shown in the top row ((a), (b), and (C)). Isl-1 (green) and c-Kit (red) are shown in the middle row ((d), (e), and (f)), and Flk-1 (green) and c-kit (red) are shown in the bottom row.

We also performed immunofluorescence staining for selected CPC markers, including Nkx2.5, Isl-1, c-kit, and Flk-1. As seen in Figure 3, there is no signal for any CPC markers on the undifferentiated mES cells (Figures 3(a), 3(d), and 3(g)). Due to the 3D nature of the hydrogel, it was difficult to clearly image the cells; thus, we employed cytospin-imaging techniques of individual cells. Additionally, Supplemental Figure 1 (see Supplementary Material available online at http://dx.doi.org/10.1155/2013/752620) shows the individual filters for each marker, and there is no signal for any CPC markers for the undifferentiated mES cells. Cells maintained in differentiation media on 2D collagen IV-coated dishes exhibited expression for Nkx2.5 and Isl-1 (Figure 3(b), supplemental Figure 2) as did the cells encapsulated in the 3D hydrogel (Figure 3(c), supplemental Figure 3). Encapsulated cells in the 3D hydrogel also displayed positive staining for c-Kit and Isl-1 (Figure 3(f)) as did those cells cultured on collagen IV in 2D conditions (Figure 3(e)). Additionally, cells cultured on both 2D collagen IV dishes and the 3D hydrogel both exhibited signals for Flk-1 and c-Kit (Figures 3(h) and 3(i)). It is notable that the cells maintained on the 2D collagen IV-coated dishes were more plentiful and were almost completely confluent by day five. Cells maintained in the hydrogel maintained a single-cell morphology, similar to that seen in Figure 1(c).

**FIGURE 4:**
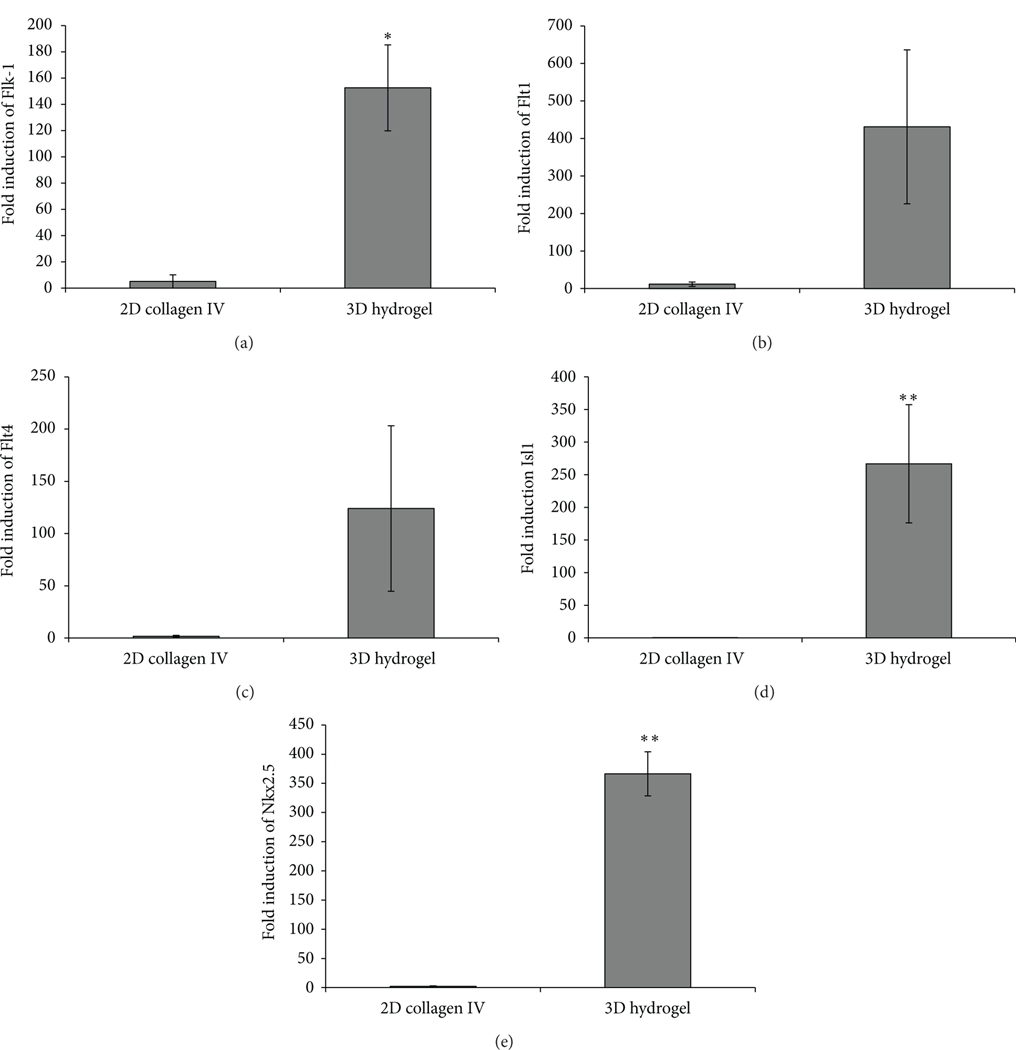
qPCR analysis. qPCR was performed on RNA collected from 2D and 3D culture conditions after five days in differentiation media. The ΔΔCt comparison method was used to calculate the fold-level expression with the undifferentiated mES cells as the reference group. CPC markers Flk-1 (a), Flt1 (b), Flt4 (c), Isl-1 (d), and Nkx2.5 (e) were evaluated. ^∗^*P* < 0.05, ^∗∗^*P* < 0.01.

To confirm the results we observed from the immunostaining and FACS analyses, we performed qPCR (Figure 4). Using undifferentiated mES v6.5 cells as our comparison group, we observed relatively no difference between undifferentiated mES cells and cells cultured on 2D collagen IV for one of the CPC markers Isl1 (Figure 4(d)). mES cells cultured in 2D conditions on collagen IV showed a higher expression for the VEGF receptor surface markers, which are known CPC markers: Flk-1 (5.06±5.01 fold expression, Figure 4(a)), Flt1 (7.85 ± 7.77 fold expression, Figure 4(b)), and Flt4 (0.23 ± 0.13 fold expression, Figure 4(c)). Additionally, we examined the cardiac development genes Nkx2.5 (2.39 ± 1.22 fold expression, Figure 4(d)) and Isl1 (0.31 + 0.04 fold expression, Figure 4(e)). However, when we encapsulated cells in the 3D hydrogel environment, we observed significantly higher expression of the two CPC markers examined. Isl1 expression was increased dramatically when encapsulated in the hydrogel (390.73 ± 70.10) compared to 2D collagen IV (0.31 ± 0.04). The other CPC marker, Nkx2.5, was also significantly increased in the 3D hydrogel (147.56 ± 37.86) compared to 2D collagen IV (2.39 ± 1.22). Additionally, we observed about 30x significantly higher expression in 3D hydrogel than the 2D collagen IV-cultured cells for Flk-1 (152.50 + 32.73 fold expression, Figure 4(a)). We also observed a marked increase in expression for Flt1 (338.35 ± 205.45fold expression, Figure 4(b)) and Flt4 (35.37 ± 30.41 fold expression, Figure 4(e)).

## 4. Discussion

The role of the ECM within a stem cell niche has recently been widely studied. However, the role of the ECM within the cardiovascular progenitor cell niche is still relatively unknown. Recently, it has been shown that the addition of ECM proteins to an *in vitro* culture contributes to the induction of a CPC population [2, 4]. Here, we demonstrate that the addition of a 3D element further enhances that induction.

Many factors are known to regulate cell-fate decisions, including growth factors, components of the ECM, neighboring cells, and the physical characteristics of the niche. The idea of a stem cell niche as a specialized microenvironment was first introduced by Schofield almost 35 years ago [1]. The stem cell niche is defined as an *in vivo* microenvironment, which regulates stem cell survival, self-renewal, and differentiation. The niche also regulates interactions between stem cells and growth factors, cell-cell contacts, and cell-matrix adhesions [9]. These interactions are extremely important to understand how stem cells not only terminally differentiate but also maintain their multipotent state while residing within the confines of the niche. The hematopoietic stem cell niche has been widely studied for many years [9]. One way to induce differentiation is to add growth factors to the culture medium: additional growth factors can be added to culture substrates [10]. The use of feeder cells in human ES cell cultures suggests that paracrine effects are equally important.

Recently, researchers have begun to examine the physical characteristics of the niche environment [7]. The ECM of the niche mediates cell-cell and cell-matrix interactions, which are extremely important to cell-fate regulation. Various ECM proteins have been used in ES cell culture. Notably, Matrigel is used commonly, which is a milieu of common ECM proteins. We have previously identified the importance of collagen IV to induce a CPC population from a murine ES cell population [2].

Additionally, the stem cell niche also provides a 3D structure to the microenvironment. Within this environment, different forces are imposed on the cells. In efforts to replicate these forces, substrates have been made of varying stiffness. Harder surfaces promote a terminal differentiation towards an osteogenic lineage [11]. Softer surfaces promote differentiation down a soft tissue lineage. Neurons, in particular, seem to respond favorably to softer surfaces with the addition of the appropriate chemical cues [12]. It has also been shown that simply incorporating a mechanistic strain to the cell culture can induce a smooth muscle differentiation pathway [13]. This type of stiffness has been observed in the cardiovascular field. Fibrotic scars that develop after myocardial infarction create local rigid surface. This can create a signal to surrounding cells and has been shown to induce bone formation in the heart [14–16]. Future work will include examining the effects of the modulus of the 3D hydrogel on the cardiovascular commitment of mES cells.

Research has turned to synthetic materials to attempt to recapitulate the native stem cell niche using tissue-engineering approaches. Cell shape has been indicated as a regulator of cell fate. In an effort to control shape, researchers have modified cell culture surfaces with various nanotopography. Cell alignment has been observed on highly porous 3D polystyrene substrates as compared to traditional 2D surfaces [17]. Alignment has also been observed along the direction of grooves formed in laminin [18]. We have also shown that incorporating nanostructures into a culture can influence cellular behavior [19]. Additionally, cellular morphology is greatly different between *in vitro* 2D culture and *in vivo* 3D native tissue, and the addition of this 3D component has become more widespread in many research groups. The study of ES cells often leads to the development of embryoid bodies, which are a 3D structure that spontaneously forms. This is further evidence of the importance of incorporating a 3D aspect into the study of stem cells.

In an effort to study the 3D effects of the niche microenvironment, we have opted to use the Hystem-C hydrogel. Glycosan is a subsidiary of BioTime, which plans to use these hydrogels in future clinical trials. Previously, we have worked with nanofibrous scaffolds. However, we observed that cells will take on average from 7 to 10 days to migrate into the interior of the scaffold. Since we wanted to evaluate a shorter time period and a true 3D microenvironment, we decided to use a hydrogel system. In particular, this hydrogel system has been used in many stem cell studies, including a recent study to determine the effects of progenitor cells delivered *in vivo* following a cardiovascular insult.

In this study, we demonstrate the ability of a hyaluronan-based 3D hydrogel to potently induce an Flk-1^+^ CPC population. We have shown that not only the addition of a ECM protein known to be present within the endogenous CPC niche does enhance the development of a CPC population but also incorporating a 3D aspect to the culture model further augments the CPC population. With the rise of stem cell therapies, developing a useful multipotent progenitor cell for potential treatments of cardiovascular disease remains elusive. Such progenitor cell populations exist in very small populations endogenously, and *in vitro* work yields low differentiation potential. This study presents an opportunity to scale up the induction of cardiovascular progenitor cells for potential therapeutic use. This study also highlights the importance of three-dimensionality when studying nonstem cell niches. The addition of a 3D element presents a more *in vivo*-like cell culture model for the laboratory setting. Future studies will include further characterization of the contributing factors of the 3D hydrogel to both maintaining multipotency of the progenitor cell population and external factors that lead to further cell differentiation.

## 5. Conclusion

We conclusively show that the addition of a 3D element to a mES cell model greatly enhances induction of an Flk-1^+^ CPC population. We have shown that cells encapsulated in a 3D system exhibit significantly higher CPC markers than cells maintained in traditional 2D cell culture systems. This development offers evidence of the importance of characterizing and further understanding the impact of the ECM on the stem cell niche.

## Supplementary Material

Supplemental Material
