# Supplementary material for: Hyaluronan-Based Three-Dimensional Microenvironment Potently Induces Cardiovascular Progenitor Cell Populations
Source: ISRN Tissue Eng. Author manuscript; Available in PMC 2021 Jan 8. (PMC7793564; doi:10.1155/2013/752620)
Supplement: Supplemental Material [file NIHMS1637442-supplement-Supplemental_Material.docx]

**Supplemental Material**

**Hyaluronan-based three-dimensional microenvironment potently induce cardiovascular progenitor cell populations**

Jessica M. Gluck^1,2^, Jennifer Chyu², Connor Delman¹, Sepideh Heydarkhan-Hagvall¹*, W. Robb MacLellan^2,3^, Richard J. Shemin¹

1. Cardiovascular Tissue Engineering Laboratory, Department of Surgery, David Geffen School of Medicine, University of California, Los Angeles

2. Department of Medicine/Cardiology, David Geffen School of Medicine, University of California, Los Angeles

3. Division of Cardiology, School of Medicine, University of Washington

* To whom correspondence should be addressed:

Sepideh Heydarkhan-Hagvall

Cardiothoracic Surgery, David Geffen School of Medicine at UCLA

10833 Le Conte Avenue, 62-151 CHS

Los Angeles, CA 90095-1741

Phone : 310-267-1885

Fax : 310-267-3590

Email : [shagvall@mednet.ucla.edu](mailto:shagvall@mednet.ucla.edu)

**Supplemental Figure1: Immunofluorescence of undifferentiated mES cells CPC markers**

**
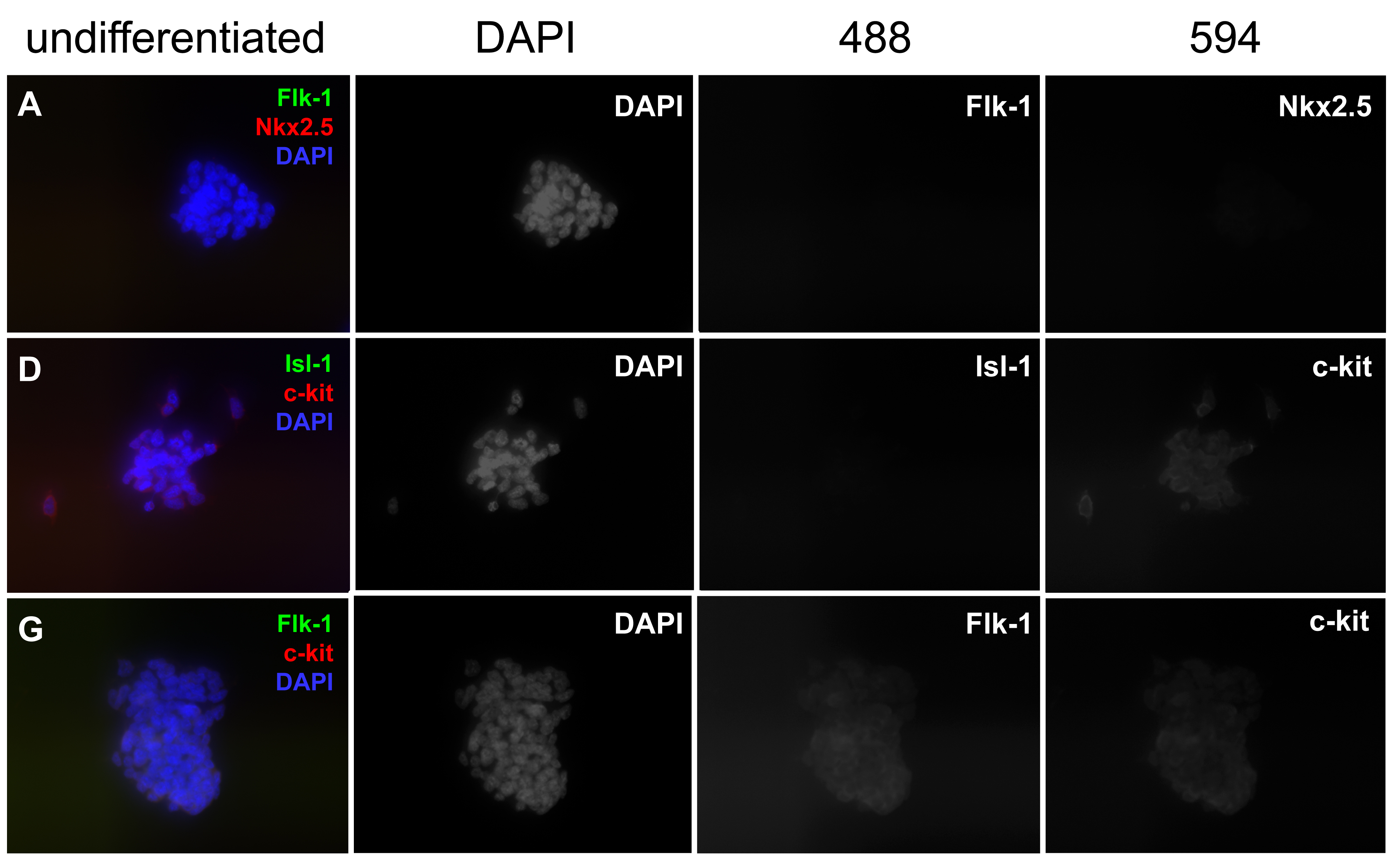
**

Individual filters of immunofluorescent images from Figure 3 (A,D,G). Original merged images are shown in the first column. Second (DAPI), third (488), and fourth (594) columns shown their respective filters. First row shows Flk-1 and Nkx2.5. Second row shows Isl-1 and c-kit. Third row shows Flk-1 and c-kit.

**Supplemental Figure 2: Immunofluorescence of 2D collagen IV CPC markers**

**
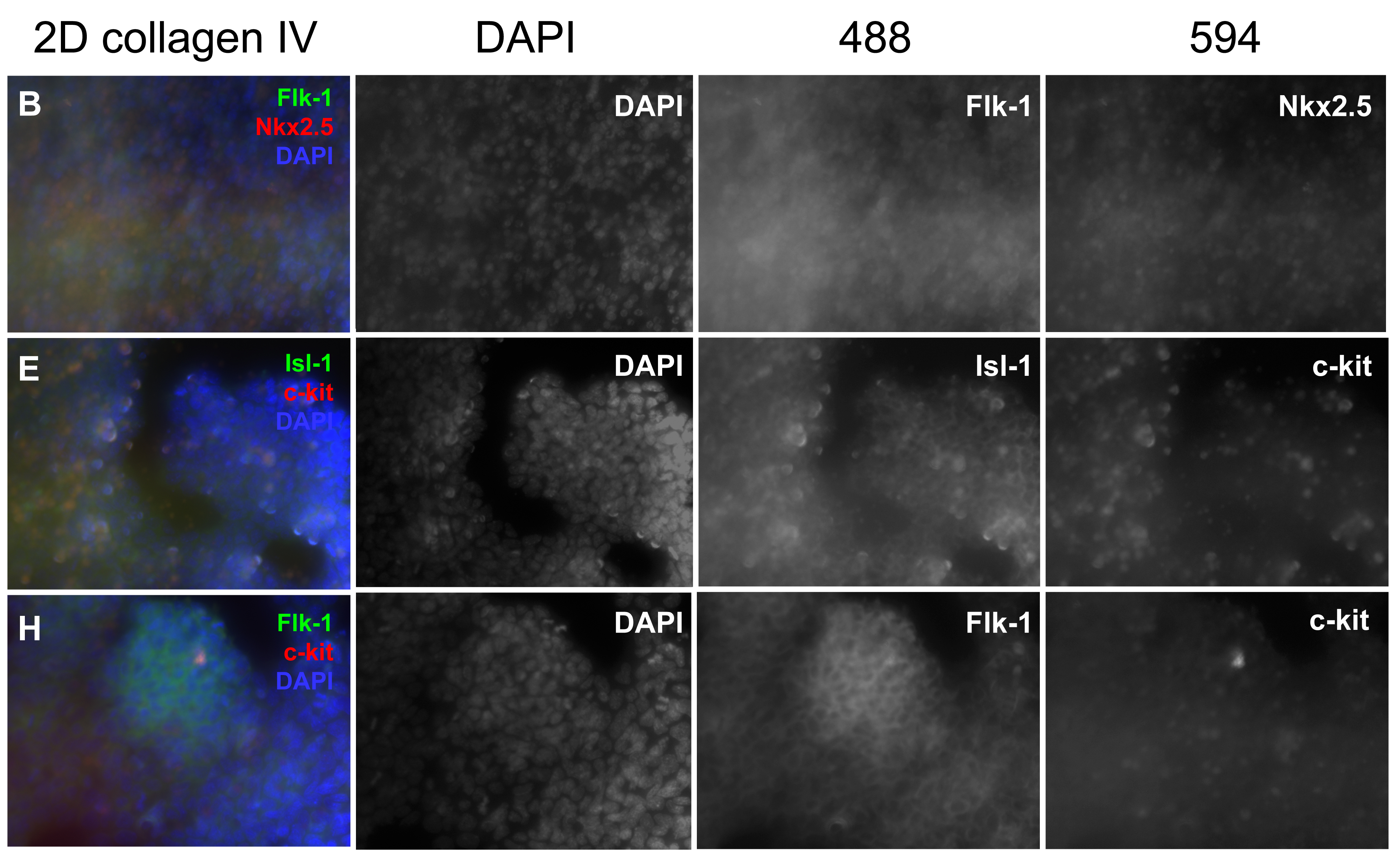
**

Individual filters of immunofluorescent images from Figure 3 (B,E,H). Original merged images are shown in the first column. Second (DAPI), third (488), and fourth (594) columns shown their respective filters. First row shows Flk-1 and Nkx2.5. Second row shows Isl-1 and c-kit. Third row shows Flk-1 and c-kit.

**Supplemental 3: Immunofluorescence of 3D Hydrogel CPC markers**


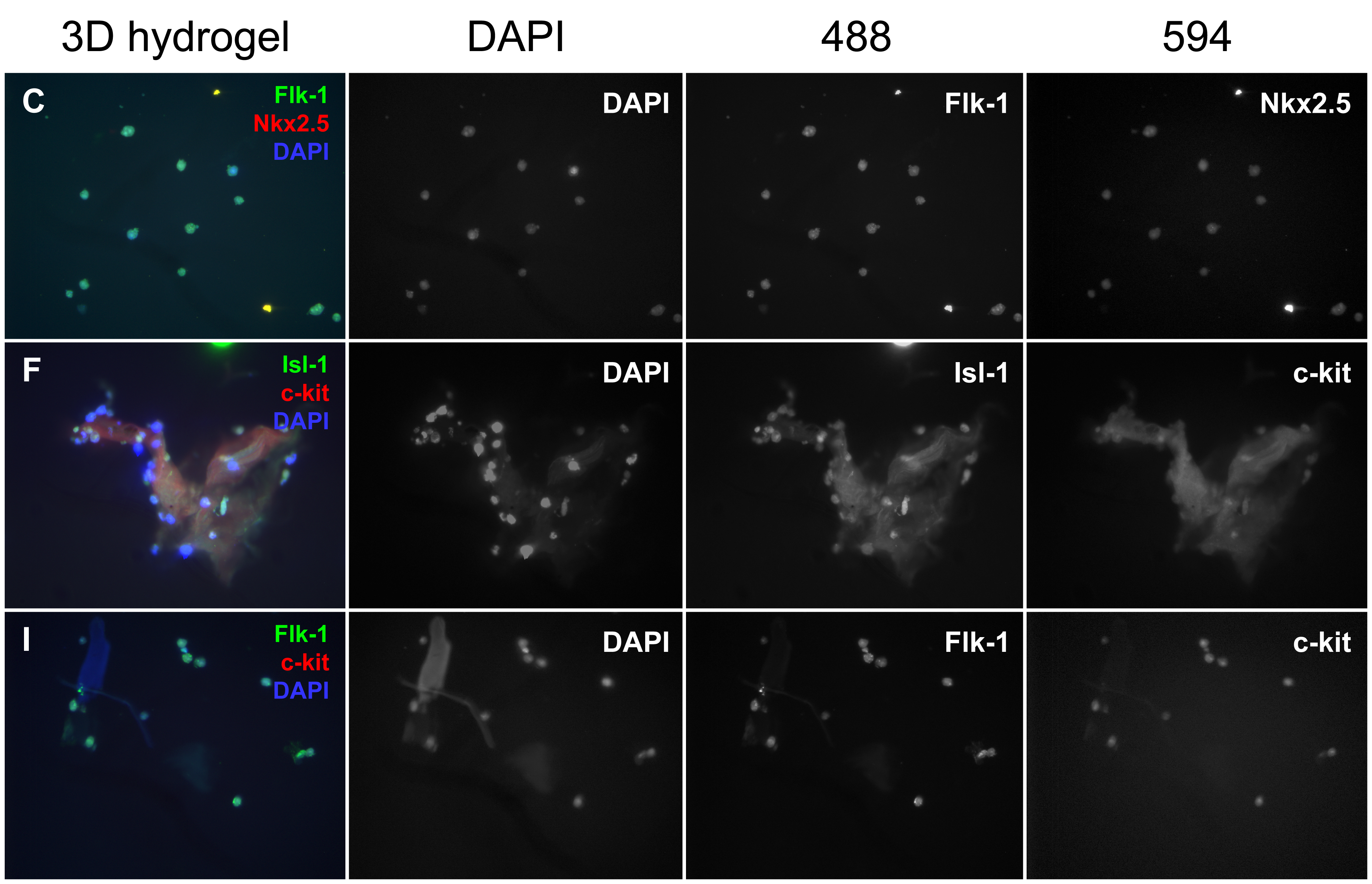


Individual filters of immunofluorescent images from Figure 3 (C,F,I). Original merged images are shown in the first column. Second (DAPI), third (488), and fourth (594) columns shown their respective filters. First row shows Flk-1 and Nkx2.5. Second row shows Isl-1 and c-kit. Third row shows Flk-1 and c-kit.
